# NLX‐112 Randomized Phase 2A Trial: Safety, Tolerability, Anti‐Dyskinetic, and Anti‐Parkinsonian Efficacy

**DOI:** 10.1002/mds.30175

**Published:** 2025-03-17

**Authors:** Per Svenningsson, Per Odin, Filip Bergquist, Karin Wirdefeldt, Dag Nyholm, Mattias Andréasson, Ioanna Markaki, Anders C. Johansson, Måns Jergil, Christopher Jankosky, Mark A. Varney, Fabienne Herbrecht, Steven A. Johnson, Adrian Newman‐Tancredi

**Affiliations:** ^1^ ASC Torsplan Stockholm Sweden; ^2^ Department of Clinical Sciences Lund University, Skåne University Hospital Lund Sweden; ^3^ Sahlgrenska Hospital Gothenburg Sweden; ^4^ Department of Pharmacology University of Gothenburg Gothenburg Sweden; ^5^ Department of Neurology Karolinska University Hospital Solna Sweden; ^6^ Uppsala University Uppsala Sweden; ^7^ Clinical Trial Consultants, AB Uppsala Sweden; ^8^ Neurolixis Inc. Park Ridge New Jersey USA; ^9^ Neurolixis SAS Castres France

**Keywords:** 5‐HT_1A_ receptor, dyskinesia, levodopa, NLX‐112, Parkinson's disease, serotonin

## Abstract

**Background:**

Levodopa‐induced dyskinesia (LID) in Parkinson's disease (PD) is associated with ‘false neurotransmitter’ release of dopamine from serotonin (5‐HT) neurons. NLX‐112 is a first‐in‐class, highly selective 5‐HT_1A_ receptor agonist which counteracts LIDs in experimental PD models.

**Objectives:**

The primary objective was to evaluate the safety and tolerability of NLX‐112 compared with placebo in people with PD. The secondary objective was to assess the preliminary efficacy of NLX‐112 in reducing LID and its effects on PD symptoms.

**Methods:**

Participants received NLX‐112 or placebo (2:1 ratio) alongside stable Parkinson's medications, with 22 participants completing the study. Dosing was up‐titrated over 28 days to 2 mg/day (1 mg twice daily), stabilized for 14 days (to day 42), and down‐titrated for 14 days. Efficacy was measured using the Unified Dyskinesia Rating Scale (UDysRS), Unified Parkinson's Disease Rating Scale (UPDRS), and Clinical Global Impression of Change (CGI‐C) following a levodopa challenge (150% of usual dose).

**Results:**

Adverse events (AEs) were mainly central nervous system (CNS)‐related and mostly occurred during up‐titration, with no serious AEs in the NLX‐112 group. There were no treatment‐induced clinically significant changes in vital signs, electrocardiogram, or laboratory parameters. NLX‐112 reduced LID from baseline levels: at day 42, UDysRS total score decreased by 6.3 points, whereas placebo group changes were not significant (−2.4). NLX‐112 also reduced parkinsonism from baseline values: UPDRS Part 3 scores decreased by 3.7 points, whereas placebo group changes were non‐significant (+0.1). In CGI‐C assessment, the NLX‐112 group showed greater improvement than the placebo group (53% vs. 29%).

**Conclusion:**

These results support further clinical investigation of NLX‐112 for treatment of PD LID. © 2025 Neurolixis SAS. *Movement Disorders* published by Wiley Periodicals LLC on behalf of International Parkinson and Movement Disorder Society.

## 1 Introduction

In Parkinson's disease (PD), conversion of levodopa to dopamine (DA) by dopaminergic neurons diminishes progressively as DA neurons are lost, so it eventually takes place in non‐dopaminergic cells and via ectopic DA release. This leads to development of abnormally regulated movements, known as levodopa‐induced dyskinesias (LIDs), which affect 30% to 50% of people with Parkinson's disease (PwP) within about 5 years of starting levodopa treatment.^1^, ^2^ Notably, serotonin (5‐HT) neurons can convert exogenous levodopa to DA, which they store and release as a ‘false neurotransmitter’ in an uncontrolled manner,^3^, ^4^, ^5^, ^6^, ^7^ influencing the number and sensitivity of post‐synaptic DA receptors, causing long‐lasting changes which underlie the development of LID.^8^, ^9^, ^10^

In animal models, LID can be reduced by inhibition of 5‐HT neurons, for example, by targeting presynaptic somatodendritic 5‐HT_1A_ autoreceptors^10^, ^11^ and/or nerve terminal 5‐HT_1B_ autoreceptors,^10^, ^12^ thus dampening ‘false neurotransmitter’ DA release. However, current serotonergic compounds are not optimized: whilst 5‐HT agonists or 5‐HT/D2 compounds such as buspirone,^13^, ^14^, ^15^ sarizotan,^16^ eltoprazine,^17^ and a buspirone/zolmitriptan combination^18^ exert anti‐dyskinetic actions, they do not improve, and can even worsen, parkinsonian symptoms.^15^, ^17^

NLX‐112 (befiradol or F13640), is a centrally acting, potent, and exceptionally selective full agonist at 5‐HT_1A_ receptors.^19^, ^20^ It potently and efficaciously reduced dyskinesia in preclinical models of LID using 6‐hydroxydopamine‐lesioned rats, and MPTP‐treated marmosets and cynomolgus macaques.^21^, ^22^, ^23^ Moreover, when tested alone, NLX‐112 also exhibited pro‐locomotor effects in 6‐OH‐DA‐lesioned rats and in MPTP‐treated marmosets, eliciting ipsilateral rotations in the former, and reducing motor disability in the latter.^21^, ^23^ In the clinic, oral NLX‐112 has previously been administered to >600 male and female subjects in safety and efficacy studies for non‐movement disorder (chronic pain) indications and has shown a good safety and tolerability profile.

Based on these results, we undertook an exploratory phase 2A, double‐blind, randomized, placebo‐controlled study of the safety, tolerability, and preliminary efficacy of NLX‐112 in PwP with troublesome LID in a levodopa challenge‐dose setting.

## 2 Methods

The study was a multicenter, randomized, double‐blind, placebo‐controlled, phase 2A trial sponsored by Neurolixis. There were five participating centers: the Center for Neurology at the Academic Specialist Center in Stockholm, Karolinska University Hospital, Skåne University Hospital, Uppsala University Hospital, and Sahlgrenska University Hospital in Gothenburg.

The study was conducted in accordance with the Declaration of Helsinki and Good Clinical Practice Guidelines and with the approval of the Swedish Medical Products Agency and the national ethical review board. PwP did not receive monetary benefits to participate in the study. The study is registered at ClinicalTrials.gov with the Identifier NCT05148884 and on the EU Clinical Trials Register with the EudraCT Number: 2020‐006053‐22.

Full inclusion and exclusion criteria are shown in Supplementary Information. Key inclusion criteria are: age 30–85 years, diagnosis of PD based on the United Kingdom Parkinson's Disease Society Brain Bank Clinical Diagnostic Criteria, and troublesome peak‐dose dyskinetic response to levodopa medication (score ≥ 1 on Part IV, Item 33 (Disability) of the Unified Parkinson's Disease Rating Scale (UPDRS) at screening (visit 1) and at day 1 (baseline, visit 2). Subject is stably and optimally treated with L‐DOPA; other anti‐PD treatments (including amantadine) are allowed if used for at least 4 weeks of previous continuous treatment. Key exclusion criteria are: history of any clinically significant disease or disorder which could either put the PwP at risk because of participation in the study, or influence the results or the PwP's ability to participate in the study; a Hoehn and Yahr (H&Y) stage of 5 when ‘OFF’; and PwP had taken an anti‐convulsant, an anti‐psychotic (except quetiapine), pindolol, tertatolol, or buspirone within 4 weeks of baseline. PwP entering the study were randomized to either NLX‐112 or to placebo groups in a 2:1 ratio to maximize information on the safety and tolerability of NLX‐112 in PwP, which was the primary endpoint of the study. The randomization list was generated by the Clinical Research Organization (CRO), Clinical Trial Consultants (CTC) AB, Uppsala, Sweden. The study utilized competitive enrollment between sites. The original randomization list was kept in a sealed envelope at the CRO and a copy at the hospital pharmacy. Sealed treatment code envelopes were kept by each site. Bottles of the study drug tablets were dispensed to the PwP at the clinics as per the randomization list.

Dose selection for NLX‐112 was based on previous clinical experience and on dose predictions from animal models of LID. NLX‐112 was up‐titrated over 28 days to a maximum of 2 mg/day (1 mg twice daily); dosing was then kept stable for 14 days and then down‐titrated over the next 14 days. Study drug and matching placebo tablets were manufactured by Sharp Clinical Services (Bethlehem, Pennsylvania, USA). PwP with intolerable adverse events (AEs) during the up‐titration period were allowed to return to the previous well‐tolerated dose at the discretion of the Investigator.

### 2.1 Primary Objective

The primary objective was to evaluate the safety and tolerability of NLX‐112 compared with placebo during 8 weeks of daily treatment in PwP with LID. Safety/tolerability assessments included: physical examination (screening and follow‐up); 12‐lead electrocardiogram (ECG, all clinic visits); vital signs (all clinic visits); frequency, intensity, and seriousness of AEs (from baseline visit through follow up visit); assessment of suicidal ideation/behavior by the Columbia Suicide Severity Rating Scale (CSSRS, all clinic visits); and clinical labs (hematology, blood chemistry, and urinalysis, all clinic visits). All subjects who were recruited into the trial were included in the Full Analysis Set (FAS) for safety and tolerability assessment.

### 2.2 Secondary Objectives

The secondary objective was to assess the preliminary efficacy of NLX‐112 in reducing troublesome LID and examine its effects on PD symptoms. The efficacy dataset included those subjects who completed the trial according to protocol (Per Protocol Set, PPS). PwP were rated for LID at baseline and on days 28 (end of up‐titration period) and 42 (end of stable dosing period). Because the level of dyskinesia experienced by PwP can vary over time, they were given 150% of their regular levodopa dose (up to a maximum of 250 mg) as a challenge 30 min before each dyskinesia assessment to improve comparability between visits. The levodopa challenge was administered 2–2.5 h after the subjects' regular morning dose or, for PwP receiving levodopa infusion, after interrupting infusion for 15–30 min. Subjects were assessed at 30, 60, and 90 min after dosing with levodopa.

LID was assessed using the Unified Dyskinesia Rating Scale (UDysRS) at three time points (30, 60, and 90 min) following levodopa challenge. Data were analyzed based on the most severe of the three scores as well as on the average score of the three time points.

PD symptoms were assessed by Unified Parkinson's Disease Rating Scale (UPDRS, classic version) within 30–90 min after levodopa challenge dosing (i.e., when the subjects were ON). Changes from baseline are shown for total score (ie, Parts 1 to 4) and individual scores for Parts 1, 2, 3, and 4.

### 2.3 Exploratory Objectives

Exploratory efficacy assessments were performed at baseline and on days 28 and 42: Clinical Global Impression of Severity (CGI‐S) and Clinical Global Impression of Change (CGI‐C), pain assessed by the King's Parkinson's Disease Pain Scale (KPPS), bladder function assessed by the International Consultation on Incontinence Questionnaire‐Overactive Bladder Module (ICIQ‐OAB), daytime sleepiness assessed by the Epworth Sleepiness Scale (ESS), mood assessed by the Hospital Anxiety Depression Scale (HADS), and quality of living with PD assessed by the Parkinson's Disease Questionnaire (PDQ‐39).

### 2.4 Statistical Analyses

For analysis of efficacy endpoints, the absolute change from baseline was analyzed using a linear mixed model (LMM). Treatment, visit (i.e., at baseline, end of up‐titration, or end of stable dosing), and the interaction treatment × visit were considered as fixed categorical effects and patient within‐treatment as a random effect. The baseline value of the dependent variable was included in the model as a continuous covariate. Visit was modeled as a repeated effect. Kenward–Roger's approximation for degrees of freedom was used. Hypotheses tests were performed at the 5% alpha level to identify between‐treatment superiority of NLX‐112 compared with placebo on the change from baseline. Furthermore, a Wilcoxon signed rank test was performed for each post‐baseline visit within each treatment (NLX‐112 or placebo) at the 5% alpha level to detect changes from baseline. No adjustment for multiple tests was performed. No formal sample size calculation was performed. A sample size of 24 PwP was deemed sufficient to evaluate the primary objective of safety and tolerability and to allow for exploratory evaluation of the secondary objectives. Data are presented in terms of arithmetic mean and standard deviation (SD). Data analyzers were blinded until after the database was locked.

## 3 Results

### 3.1 Subject Dosing and Disposition

**Figure 1:**
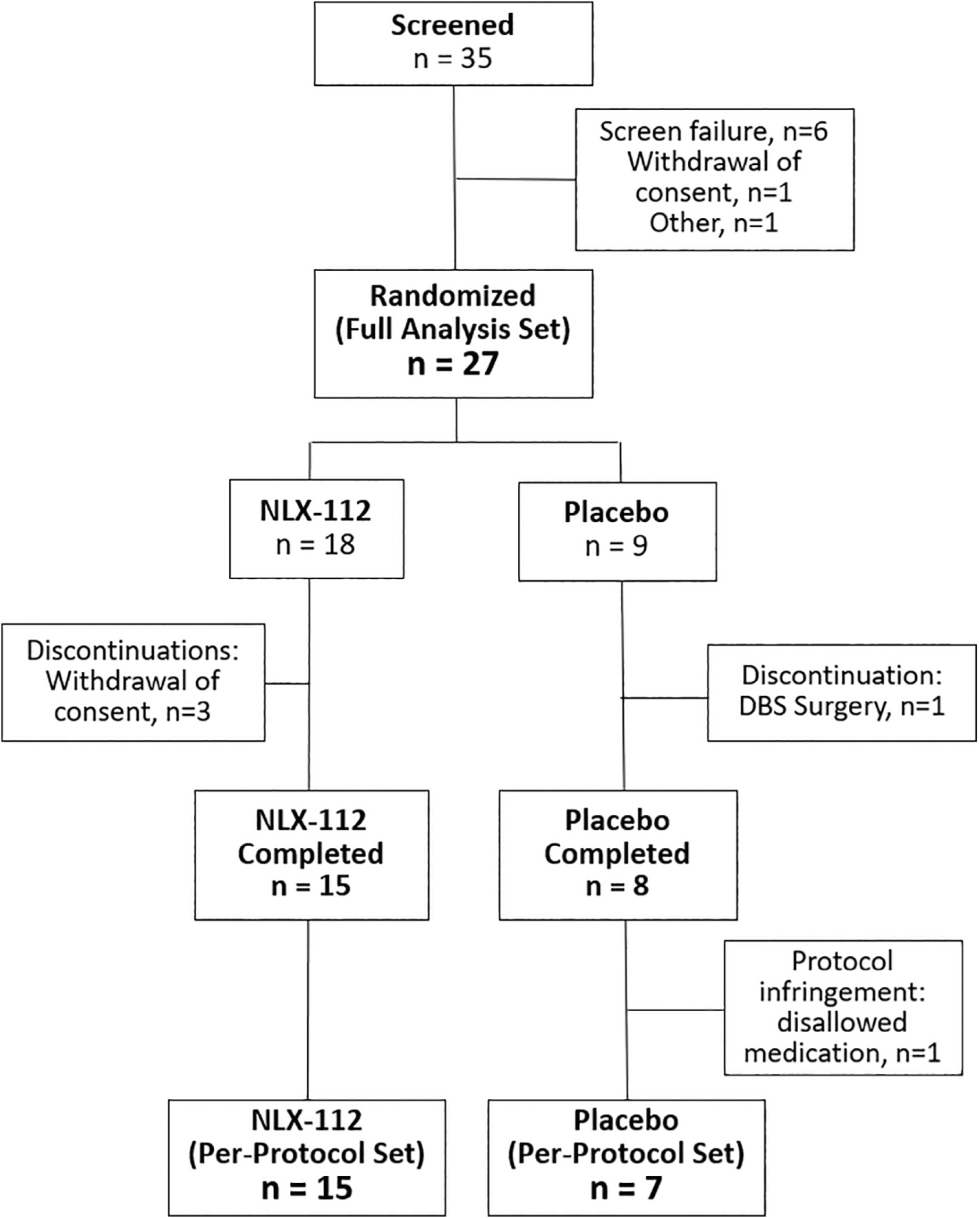
Subject disposition. DBS, deep brain stimulation.

Twenty‐seven subjects were enrolled in the study, 18 in the NLX‐112 group, and 9 in the placebo group (see Table 1 for baseline characteristics). Three subjects in the NLX‐112 group dropped out due to withdrawal of consent during the up‐titration period. There was one drop‐out in the placebo group on day 8, due to deep‐brain stimulation (DBS) surgery. One additional subject in the placebo group was excluded from the PPS because of a protocol violation (use of prohibited medication) prior to unblinding (Fig. 1). Evaluation of safety and tolerability was based on the FAS (*N* = 27) while efficacy evaluation was based on the PPS (*N* = 22). Of the subjects who completed the study (15 in the NLX‐112 group, 8 in the placebo group), most reached the highest dose of study drug (i.e., 1 mg/day twice daily): 12/15 (80%) in the NLX‐112 group and 5/8 (63%) in the placebo group. The other subjects reached at least 0.5 mg/day twice daily (see Supplementary Information). Overall, 7 subjects experienced AEs (all mild or moderate) that led to dose reductions or dose interruptions. Four of these subjects were on NLX112 and 3 on placebo.

**TABLE 1:** Demographics and disease characteristics of study subjects at baseline.

Parameter		NLX‐112 (*n* = 18)	Placebo (*n* = 9)
Age (years)	Mean (SD)	65.7 (9.7)	64.6 (6.3)
Sex	Male/female	10/8	5/4
Ethnicity	Hispanic or Latino	1	0
	Not Hispanic or Latino	17	9
Race	Asian	1	0
	White	17	9
Time since PD (years)	Mean (SD)	11.4 (4.2)	9.9 (4.6)
H&Y stage	Mean (SD)	2.41 (0.65)	2.39 (0.39)
MMSE score	Mean (SD)	29.3 (0.9)	29.1 (1.1)
LEDD (mg)	Mean (SD)	1134 (439)	1759 (1095)
UDysRS Total (Part 3 max)^a^	Mean (SD)	31.8 (8.9)	43.7 (14.8)
UDysRS Total (Part 3 average)^b^	Mean (SD)	30.4 (9.6)	42.7 (14.7)
UPDRS Total	Mean (SD)	38.3 (14.9)	42.4 (13.2)
UPDRS Part 3	Mean (SD)	17.8 (10.3)	16.9 (9.0)
On stable amantadine	Subjects	5	1^c^

*Note*: Data are shown for the Full Analysis Set (FAS).

Abbreviations: SD, standard deviation; PD, Parkinson's disease; H&Y: Hoehn and Yahr; MMSE, Mini‐Mental Status Examination; LEDD, levodopa equivalent daily dose; UDysRS, Unified Dyskinesia Rating Scale; UPDRS, Unified Parkinson's Disease Rating Scale.

^a^ UDysRS Total score, with Part 3 based on the score from the most severe of the three assessment sessions (30, 60, 90 min after levodopa dose).

^b^ UDysRS Total score, with Part 3 based on the average score of the three assessment sessions (30, 60, 90 min after levodopa dose).

^c^ This subject dropped out due to deep brain stimulation (DBS) surgery.

The study was partly conducted during the Covid‐19 pandemic. The first participant was screened on November 9, 2021 and was randomized and dosed on November 24, 2021; the last participant completed the last visit on January 18, 2023. One participant in the placebo group was diagnosed with Covid‐19 during the down‐titration period but remained in the study. For some participants, the dates of intended visits were postponed due to Covid‐19‐like symptoms; such adjustments were considered to be minor protocol deviations. A continuous risk assessment was performed, and mitigating actions were implemented to preserve PwP safety and data quality/integrity in accordance with directives from the European Medicines Agency (EMA) and local authorities.

### 3.2 Safety and Tolerability

Some 23 (85%) of the 27 randomized subjects, 16 (89%) on NLX‐112 and 7 (78%) on placebo, reported 103 AEs over the course of the study. AEs were more frequently reported during the up‐titration period in both treatment groups (see Table S1). No SAEs occurred in the NLX‐112 group. One serious adverse effect (SAE) (syncope, severe, possibly related to treatment) occurred in one subject treated with placebo, who nevertheless completed the study. Overall, there was no obvious difference in AE‐reporting frequency, severity, or causality assessments between subjects on NLX‐112 and those on placebo (Table 2).

**TABLE 2:** Adverse events occurring in >10% of people with Parkinson's disease in the NLX‐112 group

Adverse event	NLX‐112 (*n* = 18)	Placebo (*n* = 9)
Nausea	4 (22%)	0
Headache	3 (17%)	2 (22%)
Parkinsonism worsening	3 (17%)	3 (33%)
Dyskinesia worsening	2 (11%)	0
Insomnia	2 (11%)	0
Vomiting	2 (11%)	1 (14%)
Fatigue	2 (11%)	2 (22%)
Orthostatic hypotension	2 (11%)	1 (11%)
Dizziness	2 (11%)	1 (11%)
Vertigo	2 (11%)	1 (11%)
Fall	2 (11%)	0
Back pain	2 (11%)	0
Dissociation	2 (11%)	0
Restless legs syndrome	2 (11%)	0

*Note*: Table lists adverse events occurring in at least two (ie, more than 10%) subjects in the NLX‐112 group. Data are from the Full Analysis Set.

Approximately two‐thirds of the AEs (66 of 103) were assessed as possibly (53) or probably (13) related to treatment, while the remaining 37 were assessed as unlikely to be related to treatment. Most AEs (100 of 103) were assessed as mild (67) or moderate (33) in intensity. The most common AEs reported by PwP on NLX‐112 or placebo were nausea, parkinsonism, and headache (Table 2). Some 13 of 18 PwP (72%) in the NLX‐group and 6 of 9 (67%) in the placebo group had occurrences of orthostatic hypotension on one or more occasions during the study. There were no clinically significant changes from baseline in overall ECG, safety laboratory parameters, and physical examination findings and no obvious differences between the active treatment group and the placebo group in the change from baseline of these variables. There was no indication of increased suicidal ideation/behavior in any PwP during the study.

### 3.3 Anti‐Dyskinetic Actions of NLX‐112

LID symptoms were assessed at three time points following levodopa challenge (150% of usual dose). Results showed indications of anti‐dyskinetic efficacy by NLX‐112, with some within‐group and between‐group statistical significance in specific parameters. The NLX‐112 group demonstrated reductions in both the total and objective UDysRS scores on days 28 and 42 compared with baseline, while the placebo group showed no significant changes. Additionally, significant between‐group differences favored NLX‐112 when considering the average of multiple assessments (Table 3). Some participants on stable amantadine also showed improvements with NLX‐112. Five of the PwP in the NLX‐112 group were also receiving stable amantadine as part of their treatment during the trial. Four of these PwP showed decreased UDysRS total score and three showed decreased UDysRS total objective score (Parts 3 + 4) (see Supplementary Table S3 for details).

**TABLE 3:** Effects of NLX‐112 and placebo on levodopa‐induced dyskinesia and Parkinson's symptoms

	NLX‐112 (*n* = 15)			Placebo (*n* = 7)		
	Baseline score	Change vs. baseline: up‐titration (Day 28)	Change vs. baseline: stable dosing (Day 42)	Baseline score	Change vs. baseline: up‐titration (Day 28)	Change vs. baseline: stable dosing (Day 42)
UDysRS ‐ with *most severe score* of assessment sessions						
Total (Parts 1 to 4)	31.6 (9.7)	−4.1 (9.3)*	−6.3 (7.0)**	39.9 (14.2)	−2.0 (4.9)	−2.4 (9.7)
Parts 3 + 4	15.5 (6.2)	−1.7 (4.3)	−3.1 (4.2)**	18.1 (7.9)	−1.0 (1.8)	−0.1 (3.8)
Part 3 sum of items	24.7 (14.9)	−5.5 (10.4)**	−7.8 (11.1)***	28.9 (22.0)	−3.0 (3.5)	−1.7 (5.4)
UDysRS ‐ with *average score* of assessment sessions						
Total (Parts 1 to 4)	30.1 (10.5)	−4.8 (9.3)**	−6.5 (6.8)***	39.1 (13.8)	−2.1 (3.6)	−3.2 (9.0)
Parts 3 + 4	14.1 (6.6)	−2.5 (4.2)**	−3.3 (3.4)***	17.4 (7.5)	−1.1 (1.9)	−1.0 (2.9)
Part 3 sum of items	21.5 (14.4)	−5.5 (9.3)*** #	−7.1 (8.8)**** #	26.8 (20.5)	−2.29 (5.2)	−3.2 (4.4)
UPDRS						
Total (Parts 1 to 4)	37.0 (13.3)	−6.5 (7.6)***	−5.7 (6.0)**	41.3 (15.0)	−3.6 (7.0)	−3.9 (9.3)
Part 1	1.0 (1.0)	0.2 (1.5)	0.1 (1.2)	1.6 (1.5)	−0.3 (1.4)	0.3 (2.0)
Part 2	10.7 (4.4)	−2.1 (4.3)*	−1.2 (3.7)	12.9 (4.7)	−1.9 (3.2)	**−3.1 (2.0)** ^**Δ**^
Part 3	17.5 (9.4)	−3.7 (4.7)** #	−3.7 (4.6)**	17.0 (10.3)	1.3 (6.9)	0.1 (8.2)
Part 4	8.0 (2.0)	−0.9 (1.8)*	−0.9 (2.5)*	8.4 (2.0)	−2.7 (1.0)*	−1.1 (2.3)

*Note*: Results are mean (SD) of the Per‐Protocol Set for baseline and change from baseline values at days 28 (end of up‐titration period) and 42 (end of stable dosing period). Numbers in bold are significantly different to baseline: ^Δ^ *P* = 0.05, *P < 0.05, **P < 0.01, ***P < 0.001, ****P < 0.0001. Significant difference to placebo: #*P* < 0.05. Linear mixed model (LMM).

Abbreviations: UDysRS, Unified Dyskinesia Rating Scale; UPDRS, Unified Parkinson's Disease Rating Scale.

### 3.4 Anti‐Parkinsonian Actions of NLX‐112

Parkinsonian symptoms were assessed by UPDRS at baseline and on days 28 and 42. Results showed indications of antiparkinsonian efficacy by NLX‐112, with between‐group and within‐group statistically significant effects in some parameters (Table 3). Significant improvements in total UPDRS scores were observed within the NLX‐112 group compared with baseline, while the placebo group showed no significant changes. The NLX‐112 group demonstrated reductions in the total UPDRS score on days 28 and 42. For UPDRS Part 2, significant improvements were seen within the NLX‐112 group on day 28. Part 3 scores showed significantly greater decreases in the NLX‐112 group compared with the placebo group on day 28, with significant reductions within the NLX‐112 group on both days. Part 4 scores also improved significantly within the NLX‐112 group on both days, while the placebo group only showed improvement on day 28.

Of the five PwP in the NLX‐112 group that were also receiving amantadine, four showed decreased UPDRS total score and three showed decreased UPDRS Part 3 scores (see Supplementary Table S3 for details).

### 3.5 Effects of NLX‐112 on Exploratory Objectives

The NLX‐112 group showed greater improvement than the placebo group for CGI‐C: the proportion of participants (PPS dataset) in the NLX‐112 group and whose condition was rated as ‘minimally improved’, ‘much improved’, or ‘very much improved’ was 33% and 53% on days 28 and 42, respectively, compared with 29% in the placebo group on both days (Table 4). There were no consistent statistically significant effects on other exploratory objectives (see Supplementary Information).

**TABLE 4:** Effects of NLX‐112 and placebo on Clinical Global Impression of Change (CGI‐C)

CGI‐C	NLX‐112 (*n* = 15)		Placebo (*n* = 7)	
	Change vs. baseline: up‐titration (Day 28)	Change vs. baseline: stable dosing (Day 42)	Change vs. baseline: up‐titration (Day 28)	Change vs. baseline: stable dosing (Day 42)
**Total improved**	**5 (33%)**	**8 (53%)**	**2 (29%)**	**2 (29%)**
Very much improved	0	1 (6.7%)	0	0
Much improved	2 (13%)	4 (27%)	2 (29%)	1 (14%)
Minimally improved	3 (20%)	3 (20%)	0	1 (14%)
No change	9 (60%)	6 (40%)	4 (57%)	5 (71%)
Minimally worse	1 (6.7%)	1 (6.7%)	1 (14%)	0

*Note*: Table lists the number and percentage of subjects showing change from baseline at day 28 (end of up‐titration period) and at day 42 (end of stable dosing period). Numbers in bold are the sum of ‘Very much improved’, ‘Much improved’, and ‘Minimally improved’. Data are from the Per‐Protocol Set.

## 4 Discussion

The key findings of this phase 2A, randomized, double‐blind, placebo‐controlled study are that NLX‐112, a first‐in‐class, highly selective 5‐HT_1A_ receptor full agonist (1) was safe and generally well‐tolerated in PwP with troublesome LID; (2) reduced LID from baseline values, as assessed by the UDysRS; (3) reduced PD symptoms from baseline values, as assessed by UPDRS, including reduction of motor disability (UPDRS Part 3); and (4) numerically improved CGI‐C scores more than placebo‐treated PwP.

Serotonin mechanisms have long been understood to be implicated in motor fluctuations in PD, but various drug candidates exhibit limited efficacy and/or interfere with the therapeutic effects of levodopa,^15^, ^16^, ^17^, ^18^ possibly due to interaction with off‐target sites (notably antagonism of DA receptors) and/or insufficient agonist efficacy at 5‐HT_1A_ receptors. In contrast, NLX‐112 (befiradol or F13640) has exceptional selectivity and full agonist properties at 5‐HT_1A_ receptors.^19^

Overall, NLX‐112 was safe and well tolerated by most PwP in the study and no SAEs occurred in the NLX‐112 group. All AEs reported by PwP on NLX‐112 (mostly nausea, dizziness, headache, and insomnia, see Table 2) were mild to moderate in intensity, were more frequently reported during the up‐titration period (see Supplementary Table S1), and are known to be the most common treatment‐emergent AEs from previous studies on NLX‐112. In line with this favorable AE profile, there were no clinically significant changes from baseline in ECG, vital signs, safety laboratory parameters, or physical examinations in either the NLX‐112 or placebo groups. Overall, the safety profile did not differ between the NLX‐112 group and the placebo group.

The present study used an up‐titration design because as well as having high bioavailability and extended plasma exposure (half‐life >24 h), NLX‐112 is rapidly absorbed into the central nervous system (CNS). This can lead to Cmax‐related AEs, notably dizziness, headache, and nausea, which can be attenuated by a gradual up‐titration to the target dose. An alternative strategy to reduce these AEs is to use a slow‐release formulation of NLX‐112, as shown in a previous study in healthy volunteers.^24^

For anti‐LID efficacy measures, a levodopa challenge was administered at baseline and on days 28 and 42 to attenuate some of the daily variability in LID that can occur in PwP. This is useful in the context of exploratory trials with modest numbers of subjects,^17^, ^25^, ^26^ such as the present study. A reduction of UDysRS total score compared with baseline was observed in the NLX‐112 group (Table 3). Notably, the decreases in UDysRS scores in the NLX‐112 group were significant despite the short (2‐week) stable dosing period at the maximum dose. Interestingly, there was a further numerical reduction of UDysRS scores at day 42 compared with day 28, suggesting that there might be a time‐dependent effect which could accentuate with longer treatment durations. Moreover, NLX‐112 also elicited significant reductions in the sum of the item scores from UDysRS Part 3 (Objective Evaluation of Dyskinesia Impairment) compared with the placebo group, suggesting that between‐groups superiority of NLX‐112 could be demonstrated in trials which are appropriately powered for investigation of efficacy.

Amantadine, a NMDA receptor antagonist with additional pharmacological activities,^27^ is recommended for the treatment of LIDs, but many PwP either do not respond, show only marginal response,^28^ or experience loss of response over time.^29^ Moreover, amantadine is associated with unwanted side effects that can limit its utility.^30^, ^31^ The present study allowed inclusion of amantadine‐treated PwP, reflecting ‘real world’ treatment situations. In fact, the study randomized six PwP who had received amantadine for 1–11 years and they remained on this stable medication throughout the study. Five of these subjects were in the NLX‐112 group and all successfully completed the trial. Notably, four of them showed numerical reductions in the UDysRS total score and three showed reductions in UDysRS total objective score (Parts 3 + 4; see Supplementary Table S3), suggesting that NLX‐112 may provide additional benefit over amantadine for reducing LID. Nevertheless, these findings remain preliminary and need confirmation in appropriately designed trials.

Unlike previous serotonin 5‐HT_1A_ agonists, the anti‐LID activity of NLX‐112 was not accompanied by a loss of antiparkinsonian activity of levodopa. Instead, NLX‐112 further reduced parkinsonism in PwP between baseline and days 28 and 42 (UPDRS Total and Part 3 scores; Table 3). Similarly, four of the five PwP on NLX‐112 and amantadine showed numerical reductions in UPDRS scores (Table S3). These effects are notable because they occur after administration of a challenge dose of levodopa, that is, when the PwP were ‘ON’. This suggests that activation of 5‐HT_1A_ receptors by NLX‐112 elicits an additional reduction of parkinsonism through a different mechanism to that engaged by DA replacement therapy (see later comments). Animal experiments have previously shown that NLX‐112 increases rotation behavior in 6‐OH‐DA lesioned rats^23^ and reduces disability scores in MPTP‐treated marmosets,^21^ but the present observations are the first to provide an indication that such anti‐parkinsonian activity may translate to clinical use. However, in view of the small number of subjects and the short duration of the trial, more extended studies in larger cohorts of PwP are necessary to confirm these observations.

As concerns the mechanism of action of NLX‐112, its anti‐LID activity likely involves activation of raphe 5‐HT_1A_ autoreceptors and the consequent inhibition of serotonin neuron activity, thereby reducing conversion of levodopa to DA by these neurons. This is supported by experiments in 6‐OH‐DA lesioned dyskinetic rats, where NLX‐112 inhibited striatal 5‐HT release and concurrently blunted the ‘DA surge’ following levodopa administration.^23^ In contrast, the mechanisms underlying the antiparkinsonian effects of NLX‐112 require further investigation. One possibility is that NLX‐112 acts on 5‐HT_1A_ receptors in striatum, as suggested by preclinical and clinical positron emission tomography (PET) brain imaging using ^18^F‐NLX‐112.^32^, ^33^ The compound may have an inhibitory action on cholinergic interneurons,^34^ an effect which could mediate antiparkinsonian activity. Moreover, 5‐HT_1A_ receptors in rat striatum can mediate anti‐dyskinetic activity,^35^, ^36^ pointing to both raphe and striatal mechanisms in the effects of NLX‐112. Functional brain imaging and EEG investigations could shed light on this,^37^, ^38^ particularly in the context of the ‘biased agonist’ profile of NLX‐112. Indeed, NLX‐112 preferentially activates specific cellular signaling cascades (via Gαo G‐proteins)^19^ and shows accentuated activity in brain regions associated with motor control.^20^ Such biased agonist properties may underlie its superior profile compared with previous serotonergic drugs.

Some limitations should be noted. First, a difference in baseline disease severity was apparent between the NLX‐112 and placebo‐treated subjects, based on higher UDysRS and LEDD scores in the placebo group. This raises the possibility that the anti‐LID efficacy of NLX‐112 could be greater in PwP with higher initial dyskinesia scores (i.e., with a larger ‘window’ in which to show improvement). Alternatively, 5‐HT_1A_ receptor activation may be preferentially efficacious for reducing LID in subjects with less advanced PD. Either way, future studies should investigate cohorts of subjects with different disease severity scores. Second, although significant reductions from baseline values in UDysRS and UPDRS scores were observed within the NLX‐112 group, few between‐group differences from the placebo group were observed. This is likely attributable to the small cohort size and, possibly, the modest dose of NLX‐112. Hence, clear demonstration of the anti‐LID and anti‐parkinsonian properties of NLX‐112 versus placebo requires a follow‐up study with a larger sample size. Third, there were no consistent effects in measures of non‐motor symptoms (pain, anxiety/depression, sleepiness, etc.), likely because subject inclusion criteria were not based on these parameters and baseline values were generally low. Detection of potential efficacy of NLX‐112 on non‐motor symptoms requires trials conducted in PwP presenting more severe symptoms. Finally, whilst a levodopa challenge is useful in small studies to overcome variability in LID, it does not reflect normal day‐to‐day experience of PwP and is not suitable for larger studies.

In conclusion, NLX‐112 up‐titrated over 4 weeks to a fixed dose of up to 2 mg/day, maintained at that dose for a further 2 weeks, and down‐titrated over 2 weeks, was safe and well tolerated by most PwP. Moreover, NLX‐112 reduced both LID and overall PD symptoms (and parkinsonism in UPDRS Part 3) from baseline values, warranting its further investigation as a drug candidate for treatment of Parkinson's disease and related movement disorders.

## Author Roles

(1) Research Project: A. Concepualization, B. Methodology, C. Validation, D. Investigation, E. Resources, F. Data Curation, G. Visualization; (2) Statistical Analysis: A. Formal Analysis; (3) Manuscript Preparation: A. Writing of the First Draft, B. Review and Critique; (4) Other: A. Project Administration; B. Supervision, C. Funding Acquisition.

P.S.: 1B, 1C, 1D, 1E, 1F, 3A.

P.O.: 1B, 1C, 1D, 1E, 1F.

F.B.: 1B, 1C, 1D, 1E, 1F.

K.W.: 1B, 1C, 1D, 1E, 1F, 3A.

D.N.: 1B, 1C, 1D, 1E, 1F.

M.A.: 1B, 1C, 1D, 1E, 1F.

I.M.: 1B, 1C, 1D, 1E, 1F, 3A.

A.C.J.: 1B, 1C, 1D, 1E, 1F.

M.J.: 1F, 2A, 4A, 4B.

C.J.: 1A, 4A, 4B.

M.A.V.: 1A, 4A, 4B, 4C.

F.H.: 1A, 4A, 4B.

S.A.J.: 1A, 4A, 4B, 4C.

A.N.‐T.: 1A, 1G, 3A, 4A, 4B, 4C.

## Supporting information


**Data S1.** Supporting Information.

## Data Availability

Data will be made available upon reasonable request.
