# Supplementary material for: NLX‐112 Randomized Phase 2A Trial: Safety, Tolerability, Anti‐Dyskinetic, and Anti‐Parkinsonian Efficacy
Source: Mov Disord. 2025 Mar 17;40(6):1134–42. doi: 10.1002/mds.30175 (PMC12160962; doi:10.1002/mds.30175)
Supplement: Supplementary file 1 — Data S1. Supporting Information. [file MDS-40-1134-s001.docx]

# **NLX-112 for dyskinesia and parkinsonism Supplementary Information**

## **Inclusion criteria:**

1. Subject was 30 – 85 years old (inclusive) with a diagnosis of idiopathic PD according to the UK Parkinson’s Disease Society Brain Bank Clinical Diagnosis criteria.

2. PwP was stably and optimally treated with levodopa; other anti-PD treatments were allowed if used for at least 4 weeks of previous continuous treatment.

3. PwP agreed to be challenged with 150% of their normal levodopa dose (maximum levodopa dose 250 mg) 30 minutes prior to efficacy assessments at baseline (Visit 2) and at the 2 efficacy clinic visits (Visits 6 and 7).

4. PwP exhibited troublesome peak-dose LID, confirmed by a score of at least 1 on part IV, item 33 (disability) of the Unified Parkinson’s Disease Rating Scale (UPDRS) at screening (Visit 1) and at Day 1 (baseline, Visit 2).

5. At least 90 minutes in total for each 24‑hour period during 2 days were indicated as “ON with troublesome dyskinesia” (according to the PD Home Dyskinesia Diary) prior to Day 1 (baseline, Visit 2).

6. PwP (and/or caregivers) were required to demonstrate the ability to complete an electronic PD Home Dyskinesia Diary (Kinesia 360 system), with concordance in ON time with dyskinesia between study staff and PwP. Two consecutive 24-hour diaries were completed in the week prior to clinic visits at baseline and on days 28 and 42

7. PwP could read well enough to understand the informed consent document and other patient materials.

8. Female PwP of child-bearing potential had to have a negative urine pregnancy test at screening (Visit 1) and on Day 1 (Visit 2), had to agree to avoid pregnancy during the study, had to practice abstinence (only allowed when this is the preferred and usual lifestyle of the patient) or had to agree to use a highly effective method of contraception with a failure rate of < 1% to prevent pregnancy (combined [estrogen and progestogen containing] hormonal contraception associated with inhibition of ovulation [oral, intravaginal, transdermal], progestogen-only hormonal contraception associated with inhibition of ovulation [oral, injectable, implantable], intrauterine device [IUD] or intrauterine hormone-releasing system [IUS]) starting from 4 weeks prior to administration of the study drug and continuing during the course of the study until 4 weeks after the last investigational medicinal product (IMP) administration. Female PwP had to agree to refrain from donating eggs from the date of dosing until 3 months after dosing with the IMP. Their male partner had to agree to use a condom during the same time frame if he had not undergone vasectomy.

Females of non-childbearing potential were defined as pre-menopausal females who were sterilized (tubal ligation or permanent bilateral occlusion of fallopian tubes); or females who had undergone hysterectomy or bilateral oophorectomy; or post-menopausal defined as 12 months of amenorrhea (in questionable cases a blood sample with detection of follicle stimulating hormone [FSH] 25-140 IE/L was considered confirmatory).

Male PwP had either to be vasectomized, consent to use condom or practice sexual abstinence to prevent pregnancy and drug exposure of a partner and refrain from donating sperm from the date of dosing until 3 months after dosing with the IMP. Their female partner of child-bearing potential had to use highly effective contraceptive methods with a failure rate of < 1% to prevent pregnancy (see above) during the same period.

Note: PwP who used continuous intestinal levodopa infusion were allowed. For levodopa challenge, they were required to stop the infusion for 15-30 minutes before the administration of an oral levodopa dose equivalent to 300% of their hourly infusion levodopa equivalent dose. The time of the day and the time between stopping the pump and administering the oral dose were the same on all assessments

## **Exclusion criteria:**

1. PwP had severe PD with a Hoehn and Yahr stage = 5.

2. PwP had unstable medical status, prior brain surgery against tumors or hemorrhage (excluding deep brain stimulation [DBS], *i.e.,* DBS patients were allowed to be enrolled) or was scheduled to receive surgery during the trial period.

3. PwP had orthostatic hypotension: a decrease in systolic blood pressure (at least 20 mm Hg) or diastolic blood pressure (at least 10 mm Hg) within 3 minutes of the patient standing up, compared to pressures obtained while in a sitting position for at least 5 minutes. At screening and baseline visits (Visit 1 and Visit 2), vital signs to assess orthostatic hypotension were conducted in triplicate, 15‑20 minutes apart, with the average of the 3 assessments used for exclusion.

4. PwP had dementia (Mini Mental Status Exam [MMSE] <20).

5. PwP had clinically significant renal or liver disorder.

6. PwP currently exhibited generalized obsessive-compulsive disorder, panic disorder, bipolar disorder, post-traumatic stress syndrome (PTSD), clinically significant parasomnias or any other psychotic disorder as established by structured clinical interview for DSM disorders (SCID). Visual hallucinations were allowed.

7. Any suicidal actions in the past 2 years (per investigator judgement *i.e.,* actual attempt, interrupted attempt, aborted attempt, or preparatory acts or behavior).

8. Any suicidal ideation of type 4 or 5 in the Columbia Suicide Severity Rating Scale (C-SSRS) in the past 3 months (*i.e.,* active suicidal thought with intent but without specific plan, or active suicidal thought with plan and intent).

9. PwP had taken an anti-convulsant, an anti-psychotic (except quetiapine), pindolol, tertatolol or buspirone within 4 weeks of baseline (Day 1, Visit 2).

10. PwP had taken, within 4 weeks of baseline (Day 1, Visit 2), any medication that inhibits or up-regulates CYP450 3A4.

11. PwP had concurrently participated in another investigational drug trial or had participated in another investigational drug trial within the past 3 months.

12. PwP was at high risk of non-compliance in the Investigator’s opinion.

## **Dose titration schedule**

**Up-titration period (4 weeks):**

Days 1-4 (0.25 mg/day): 1 tablet in the morning, none in the evening

Days 5-8 (0.5 mg/day): 1 tablet in the morning and 1 tablet in the evening

Days 9-12 (0.75 mg/day): 2 tablets in the morning, 1 tablet in the evening

Days 13-16 (1.0 mg/day): 2 tablets in the morning, 2 tablets in the evening

Days 17-20 (1.25 mg/day): 3 tablets in the morning, 2 tablets in the evening

Days 21-24 (1.5 mg/day): 3 tablets in the morning, 3 tablets in the evening

Days 25-28 (1.75 mg/day): 4 tablets in the morning, 3 tablets in the evening

**Constant dose period (2 weeks):**

Days 29-42 (2 mg/day): 4 tablets in the morning, 4 tablets in the evening

**Down-titration period (2 weeks, down-titration by 0.25 mg/day every 2 days):**

Days 43-44 (1.75 mg/day): 4 tablets in the morning, 3 tablets in the evening

Days 45-46 (1.5 mg/day): 3 tablets in the morning, 3 tablets in the evening

Days 47-48 (1.25 mg/day): 3 tablets in the morning, 2 tablets in the evening

Days 49-50 (1.0 mg/day): 2 tablets in the morning, 2 tablets in the evening

Days 51-52 (0.75 mg/day): 2 tablets in the morning, 1 tablet in the evening

Days 53-54 (0.5 mg/day): 1 tablet in the morning and 1 tablet in the evening

Days 55-56 (0.25 mg/day): 1 tablet in the morning, none in the evening

PwP in the placebo arm followed the same dose escalation, constant dose and down-titration schedule, but took the matching placebo tablets. PwP with intolerable adverse events (AEs) during the 28-day up-titration period were allowed to return to the previous tolerated dose at the discretion of the Investigator, or they could withdraw from the study if they so chose.

For additional information on the dose-titration, see Supplementary Figure S1 below.

# **PD Home Dyskinesia eDiary results**

Based on time recorded in the PD Home Dyskinesia eDiary, the proportion of ‘good ON time’ experienced by PwP at baseline was 57.6% in the NLX-112 group (n=15) and 54.8% in the placebo group (n=7).

In the NLX-112 group, mean absolute change from baseline in good ON time was +5.7% (n=13) at day 28 and +5.2% (n=12) at day 42.

In the placebo group, mean absolute change from baseline in good ON time was +2.5% (n=6) at day 28 and +3.4% (n=6) at day 42.

The results show that PwP in the NLX-112 group experienced a greater numerical increase in ‘good ON time’ than those in the placebo group but there were no statistically significant changes from baseline within or between the treatment groups.

Compliance was lower on days 28 and 42 for both groups, compared to baseline.

**Figure S1: Dose Titration Schedule and Maximum Doses Reached by Study Participants**


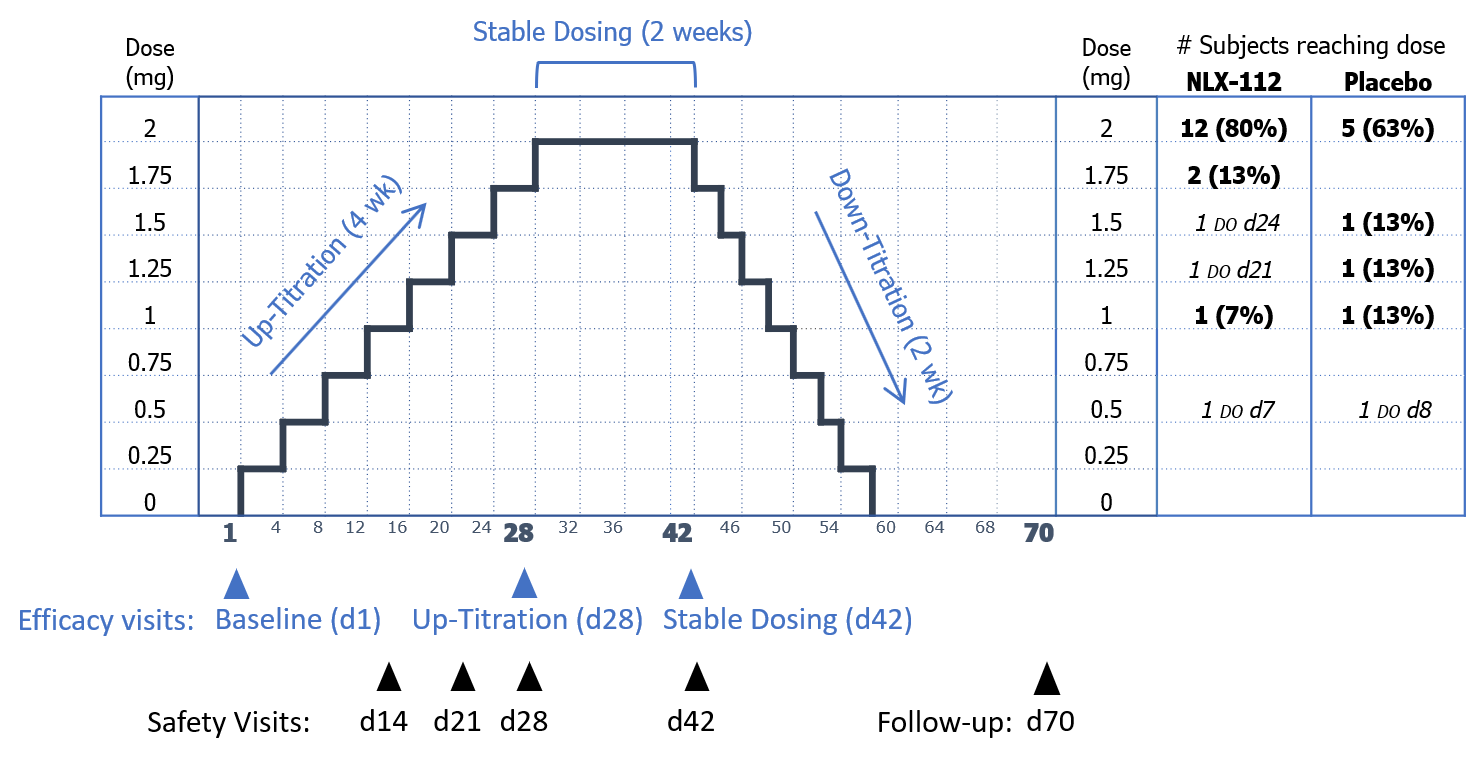


Dosing of study drug was up-titrated every 4 days by 0.25 mg to a maximum of 2 mg (or the best-tolerated dose) by day 28. Dosing was then maintained stable for 2-weeks (up to day 42) and down-titrated in 0.25 mg steps every 2 days until day 56. Efficacy visits took place at baseline and days 28 and 42. Safety visits took place at days 14, 21, 28, 42 with a follow-up at day 70 (2 weeks after last dose). Columns on the right indicate the number of subjects reaching each dose (Full Analysis Set). Numbers in bold with percentages are for subjects who completed the study (NLX-112 n=15; Placebo n=8). DO: subject dropped-out at indicated day.

# **Table S1: Adverse Events by treatment period (Full Analysis Set)**

| **System organ class  Preferred term** | **NLX-112  Up-Titration (N=18)** | | **NLX-112  Stable Dosing (N=15)** | | **NLX-112  Down-Titration (N=15)** | | **Placebo  Up-Titration (N=9)** | | **Placebo  Stable Dosing (N=8)** | | **Placebo  Down-Titration (N=8)** | | **Follow-up (N=27)** | | **Total (N=27)** | | |
| --- | --- | --- | --- | --- | --- | --- | --- | --- | --- | --- | --- | --- | --- | --- | --- | --- | --- |
|  | n (%) | m | n (%) | m | n (%) | m | n (%) | m | n (%) | m | n (%) | m | n (%) | m | n (%) | m |  |
| **Total ^#^** | **14 (78%)** | **52** | **8 (53%)** | **11** | **4 (27%)** | **6** | **7 (78%)** | **17** | **4 (50%)** | **7** | **4 (50%)** | **7** | **3 (11%)** | **3** | **23 (85%)** | **103** |  |
| Dizziness | 2 (11%) | 2 | 0 | 0 | 0 | 0 | 1 (11%) | 1 | 0 | 0 | 0 | 0 | 0 | 0 | 3 (11%) | 3 |  |
| Dyskinesia | 2 (11%) | 2 | 0 | 0 | 0 | 0 | 0 | 0 | 0 | 0 | 0 | 0 | 0 | 0 | 2 (7.4%) | 2 |  |
| Headache | 2 (11%) | 2 | 1 (6.7%) | 1 | 1 (6.7%) | 1 | 2 (22%) | 2 | 0 | 0 | 1 (13%) | 1 | 0 | 0 | 5 (19%) | 7 |  |
| Parkinsonism | 3 (17%) | 3 | 0 | 0 | 0 | 0 | 3 (33%) | 5 | 1 (13%) | 1 | 1 (13%) | 1 | 0 | 0 | 6 (22%) | 10 |  |
| Restless legs syndrome | 1 (5.6%) | 1 | 1 (6.7%) | 1 | 0 | 0 | 0 | 0 | 0 | 0 | 0 | 0 | 0 | 0 | 2 (7.4%) | 2 |  |
| Dissociation | 1 (5.6%) | 1 | 1 (6.7%) | 1 | 0 | 0 | 0 | 0 | 0 | 0 | 0 | 0 | 0 | 0 | 2 (7.4%) | 2 |  |
| Insomnia | 1 (5.6%) | 1 | 0 | 0 | 1 (6.7%) | 1 | 0 | 0 | 0 | 0 | 0 | 0 | 0 | 0 | 2 (7.4%) | 2 |  |
| Nausea | 2 (11%) | 2 | 1 (6.7%) | 1 | 1 (6.7%) | 1 | 0 | 0 | 0 | 0 | 0 | 0 | 0 | 0 | 4 (15%) | 4 |  |
| Vomiting | 2 (11%) | 2 | 0 | 0 | 0 | 0 | 1 (11%) | 1 | 0 | 0 | 0 | 0 | 0 | 0 | 3 (11%) | 3 |  |
| Back pain | 1 (5.6%) | 1 | 0 | 0 | 1 (6.7%) | 1 | 0 | 0 | 0 | 0 | 0 | 0 | 0 | 0 | 2 (7.4%) | 2 |  |
| Fatigue | 1 (5.6%) | 1 | 1 (6.7%) | 1 | 0 | 0 | 1 (11%) | 1 | 2 (25%) | 2 | 0 | 0 | 0 | 0 | 4 (15%) | 5 |  |
| Orthostatic hypotension | 0 | 0 | 2 (13%) | 2 | 0 | 0 | 0 | 0 | 1 (13%) | 1 | 0 | 0 | 0 | 0 | 3 (11%) | 3 |  |
| Vertigo | 2 (11%) | 2 | 0 | 0 | 0 | 0 | 1 (11%) | 1 | 0 | 0 | 0 | 0 | 0 | 0 | 3 (11%) | 3 |  |
| Fall | 1 (5.6%) | 2 | 1 (6.7%) | 1 | 0 | 0 | 0 | 0 | 0 | 0 | 0 | 0 | 0 | 0 | 2 (7.4%) | 3 |  |

^#^ Text in bold type (first line) shows total AEs occurring in the whole subject population. Subsequent lines show AEs occurring in at least 2 (i.e., >10%) of the subjects in the NLX-112 group.

# **Table S2: Effects of NLX-112 and Placebo on measures of non-motor symptoms (Per-Protocol Set)**

|  | **NLX-112 (n=15)** | | | **Placebo (n=7)** | | |
| --- | --- | --- | --- | --- | --- | --- |
|  | **Baseline score** | **Change vs Baseline:**  **Up-Titration (d28)** | **Change vs Baseline:**  **Stable Dosing (d42)** | **Baseline  Score** | **Change vs Baseline:**  **Up-Titration (d28)** | **Change vs Baseline:**  **Stable Dosing (d42)** |
| **ESS** | 8.3 (5.2) | -1.1 (3.3) | -0.9 (3.4) | 9.0 (4.7) | 2.3 (3.7) | 1.4 (4.2) |
| **HADS (Anxiety)** | 4.5 (2.5) | -0.5 (2.5) | -0.3 (3.6) | 4.4 (3.4) | -0.1 (1.7) | 1.0 (2.8) |
| **HADS (Depression)** | 3.7 (2.7) | -0.4 (1.7) | -0.6 (1.9) | 3.0 (1.8) | 0.0 (2.4) | 0.4 (2.4) |
| **ICIQ-OAB** | 18.3 (11.7) | 0.7 (6.5) | -0.8 (8.02) | 14.3 (14.7) | 4.1 (5.4) | **6.9 (9.2)*** |
| **KPPS** | 16.0 (9.9) | **-4.6 (7.5)**** | **-4.8 (7.8)**** | 24.0 (20.9) | -5.9 (6.8) | **-6.9 (6.7)*** |
| **PDQ39** | 18.7 (10.2) | -1.5 (8.0) | -0.6 (6.5) | 23.5 (11.8) | -1.7 (5.1) | -1.3 (7.7) |

|  |
| --- |

Data are mean (S.D.) from the Per-Protocol Set for Baseline values and change from baseline values at day 28 (end of up-titration period) and day 42 (end of stable dosing period). Numbers in bold are significantly different to baseline: *p<0.05, **p<0.01. Linear mixed model (LMM). ESS: The Epworth Sleepiness Scale; HADS: The Hospital Anxiety Depression Scale; ICIQ-OAB: International Consultation on Incontinence Questionnaire – Overactive Bladder Module; KPPS: King’s Parkinson’s disease Pain Scale; PDQ39: The Parkinson’s Disease Questionnaire.

# **Table S3: Effects of NLX-112 on LID and parkinsonism in subjects treated with amantadine**

| Sex/Age | Amantadine  Treatment Duration (years) | UDysRS Total | | | UDysRS Parts 3+4 | | UPDRS Total | | UPDRS Part 3 | | |
| --- | --- | --- | --- | --- | --- | --- | --- | --- | --- | --- | --- |
|  |  | **Baseline score** | | **Change vs Baseline (d42)** | **Baseline score** | **Change vs Baseline (d42)** | **Baseline score** | **Change vs Baseline (d42)** | **Baseline score** | | **Change vs Baseline (d42)** |
| F/51 | 1 | 18 | | **-6** | 11 | **-6** | 22 | **-5** | 8 | | **-4** |
| M/69 | 2 | 43 | | **-2** | 22 | +3 | 44 | **-15** | 22 | | **-9** |
| F/75 | 7 | 44 | | +7 | 29 | +2 | 51 | **-11** | 28 | | **-13** |
| F/49 | 11 | 39 | | **-11** | 28 | **-11** | 26 | **-5** | 11 | | +3 |
| F/72 | 6 | 31 | | **-8** | 17 | **-6** | 30 | +3 | 15 | | +2 |
| **Mean Change vs Baseline** | | |  | **-4.0** |  | **-4.4** |  | **-6.6** |  | **-4.2** | |

Table shows UdysRS and UPDRS scores at baseline and at day 42 (end of stable dosing period) for 5 subjects in the NLX-112 who were on stable concomitant amantadine. These subjects all completed the trial.
One other subjects on amantadine was in the placebo group but dropped out due to Deep Brain Stimulation surgery. Numbers in bold type indicate improvement from baseline values. The mean Change from Baseline of the UdysRS and UPDRS scores for NLX-112 / amantadine subjects is comparable to that seen for the complete NLX-112 group (see Table 3 in main manuscript).
